# Serum prostate specific antigen is a good indicator of prostatic volume in men with benign prostatic hyperplasia

**DOI:** 10.4102/phcfm.v14i1.3736

**Published:** 2022-12-15

**Authors:** Ebenezer Abotsi, Kekeli K. Adanu, Eyram C. Bansah

**Affiliations:** 1 Department of Obstetrics and Gynaecology, Ho Teaching Hospital, Ho, Ghana; 2 Department of Surgery, School of Medicine, University of Health and Allied Sciences, Ho, Ghana; 3 Department of Surgery, Richard Novati Catholic Hospital, Sogakope, Ghana

**Keywords:** correlation, prostate volume, symptom severity, PSA, benign prostatic hyperplasia

## Abstract

**Background:**

Benign prostatic hyperplasia (BPH) is the most common cause of bladder outlet obstruction in men over the age of 50 years. An association between the prostate specific antigen (PSA), International Prostate Symptoms Score (IPSS) and prostate volume (PV) may be instrumental in determining patients who may benefit from treatment. Targeted therapy will reduce the cost of care because it is unwise to treat all men with prostate enlargement to prevent complications when the risk of occurrence is negligible.

**Aim:**

To determine the correlation between the PSA, IPSS and PV in men of African descent.

**Setting:**

This was a cross sectional analysis involving 92 patients diagnosed as having symptomatic BPH at the Ho Teaching Hospital.

**Methods:**

The data were collected using standardised questionnaires. The IPSS determined urinary symptom severity. The PV was determined using a transabdominal ultrasound machine. Serum PSA was retrieved from the electronic medical records.

**Results:**

The mean PV was 61.04 cm^3^ ± 21.95 cm^3^, the mean PSA was 4.21 ng/mL ± 3.85 ng/mL, and mean IPSS of 21.59 ± 3.78. The Pearson’s correlation between PV and PSA was 0.283 (*p* = 0.01), between PV and IPSS was 0.108 (*p* = 0.30), and finally, between Serum PSA and IPSS Score was −0.086 (*p* = 0.42).

**Conclusion:**

This study showed that serum PSA has a positive correlation with PV. However, IPSS had no significant association with PSA or PV in patients with BPH.

**Contribution:**

This study provides insights into the implications of clinical parameters on the management of prostate enlargement.

## Introduction

Benign prostatic hyperplasia (BPH) is the most common cause of bladder outlet obstruction in men over the age of 50 years and worsens with age without treatment.^1^ The development of BPH is characterised by the proliferation of both stromal and epithelial cells in the transitional zones and the periurethral glands. As population ages, the incidence and the prevalence of BPH also increase. It is estimated that approximately 20% of men in their forties and 50% of men above the age of 50 will have histological evidence of BPH, with this number increasing to greater than 80% by the eighth decade.^2,3,4^ The consequences of prostate enlargement include the development of lower urinary tract symptoms (LUTS), urinary tract infection, bladder calculus and haematuria and in more advanced cases, acute urinary retention.^3^ The prostate specific antigen (PSA) since its emergence in the 1980s has revolutionised the management of prostate conditions.^5^ The transrectal ultrasound (TRUS) has also become a crucial modality in the evaluation of prostatic diseases since it was first introduced. Takahashi and Ouchi were the first to describe the use of TRUS for prostate evaluation in 1963.^6^ They employed a 3.5 MHz transducer to get the first clinically useful pictures of the prostate in 1971. Low cost, availability, no risk of radiologic contrast and radiation exposure have made it a very important tool in medical imaging. Today, TRUS is a routine tool in urology and is nearly like an extension of the urologist’s finger. Transrectal ultrasound is more sensitive to prostatic volume (PV) and other prostate characteristics.^7^ On ultrasonography, the prostate gland usually appears as a homogeneous ovoid structure with mixed low-level echoes. The severity of BPH can be assessed using the International Prostate Symptom Score (IPSS) card, a validated and standardised instrument used in this endeavour. An IPSS score > 7 is significant, warranting some form of intervention.^8^ Several studies conducted elsewhere have investigated the correlation between prostate size, PSA and symptoms score with conflicting results.^9,10,11^ Whilst some studies reported strong correlations between these parameters,^12^ others reported otherwise.^13^ Are these correlations any different in men of African descent? A study on the phenomena in Ghanaian men^14^ found a weak correlation between these parameters. However, the findings were limited by the fact that it was conducted among a general pool of men afflicted by various conditions and not just men presenting with BPH. Another study conducted in Nigeria among men reporting with various prostate conditions^15^ failed to find any correlation between the PV and IPSS. Clearly, there is the need for updated data on the subject among BPH patients of African descent.

This condition can be debilitating as men grow, it can result in significant psychological distress and advanced cases can cause urine retention. Pietrzyk and colleagues^16^ reported depressive symptoms in 22% of a large Polish cohort. They also found that these depressive symptoms were associated with the severity of LUTS, sedentary lifestyle and erectile dysfunction. The treatment is also associated with high economic costs. For instance, the management of acute urinary retention, which is one of its common complications, results in a high cost of care. Teoh et al.^17^ estimated the cost of hospital admission for an average patient with urine retention at USD2155.00 with the cost of surgery adding a further USD5000.00 to the bill. In a similar study reported by Ikuerowa and colleagues in Nigeria,^18^ USD58 800.00 was spent per annum in the management of patients with this condition. The aim of this study was to determine the correlation between these factors in men of African descent (PSA, IPSS and PV) at baseline. This can help to predict and target the subpopulation of BPH patients who are likely to suffer complications for prompt medical and surgical intervention. In addition, targeted therapy will reduce the cost of care because it is economically unwise to treat all men with prostate enlargement to prevent complications when the risk of occurrence is negligible. This will go a long way in getting to individualise management modalities and improve healthcare delivery to BPH patients.

## Research methods and design

### Study design and setting

This cross-sectional analysis was conducted among 92 patients at the urology department of the Ho Teaching Hospital, a public Teaching Hospital in Ghana. The urology department attends to an average of 5000 urological cases every year, with a significant proportion being BPH cases. Data were stored in an automated storage and retrieval system with highest assurances of patients’ privacy and confidentiality.

### Study population and sample size determination

The study comprised men between 40 and 89 years of age, diagnosed as having clinical BPH and consented to participate. The study commenced on 03 January and ended on the 20 August 2021. A total of 32 men with co-existing medical conditions such as urethral stricture, prostate carcinoma and painful anal conditions such as thrombosed anal haemorrhoids, fissures and stenosis were excluded. Sample size was determined to be 92 using the Cochran’s formula, relying on a previous study by Yeboah,^19^ at 95% confidence interval with an error margin of 0.1, a reliability co-efficient (*Z*-value) of 1.96 and a prevalence rate of 62% for BPH:


$$n=\frac{Z_{1-\frac{\alpha }{2}}^{2}P(1-P)}{MOE^{2}}$$ [Eqn 1]

Where: *n* = sample size, *P* = 62%, margin of error (MOE) = 0.1, and *Z* = reliability co-efficient (*Z*) = 1.96, *α* = 0.05.

### Sampling strategy and data collection

Data were collected by consecutive (convenience) sampling, using face-to-face interviews. A structured questionnaire was used to collate data on socio-demographic factors, LUTS, trans-abdominal ultrasonography determined PV and serum PSA. The IPSS is routinely used in our unit for the assessment of storage and voiding symptoms. It was used for assessing the four voiding symptoms of BPH. These were incomplete bladder emptying, intermittency, weak stream and straining. In assessment of the three storage symptoms, questions were asked to determine the severity of frequency, urgency and nocturia. A symptom is scored from 0 to 5 and the maximum score is 35. In the interpretation of IPSS values, Mild is assigned for scores between 0 and 7; Moderate for scores from 8 to 19; and Severe is assigned when an assessment of both storage and voiding symptoms provides a score of 20–35. The PV was estimated using the (Guangzhou, China) machine with 2 MHz convex and 5 MHz linear probes at a bladder volume of about 200 mL. Prostate volume was then calculated using the ellipsoid formula π/6 × length × breadth × height, after capturing the prostate in the mid-sagittal and transverse planes. Serum PSA was retrieved from the electronic medical records. Quality control measures instituted prior to data collection included pre-testing of questionnaires among healthy males (no known medical condition) and validation and daily entry of completed questionnaires.

### Data analysis

The data were analysed using (Atlanta, USA) and presented as tables and graphs with measures of central tendency (means and medians) and dispersion (standard deviation and interquartile ranges). Pearson correlation coefficient assessed for the inter-relationship between the study variables.

### Ethical considerations

Ethical approval was sought from the Research and Ethics Committee (REC) of the University of Health and Allied Sciences (Reference number: UHAS-REC A.12 [100] 20-21). The procedures followed were in accordance with the declaration of Helsinki.

## Results

### Background characteristics

In all, 92 patients participated with a response rate of 100%. Participants’ ages ranged between 48 and 83 years with a mean age of 65.7. Most respondents (53.3%) were unemployed and 40.2% had secondary education. Table 1 outlines the background characteristics of respondents.

**TABLE 1:** Background characteristics of respondents.

Characteristic	Frequency	%
**Age in years**		
40–49	5	5.4
50–59	18	19.6
60–69	40	43.5
70–79	22	23.9
80–89	7	7.6
**Educational attainment**		
No education	7	7.6
Primary education	9	9.8
Secondary education	37	40.2
Tertiary education	39	42.4
**Employment status**		
Employed	43	46.7
Unemployed	49	53.3
**Marital status**		
Single	5	5.4
Married	61	66.3
Divorced	16	17.4
Widowed	10	10.9
**Religion**		
Christian	67	72.8
Muslim	22	23.9
Others	3	3.3
**Health insurance**		
Yes	87	94.6
No	5	5.4

Out of the 92 participants, the mean PV was 61.04 cm^3^ ± 21.95 cm^3^, and a positive skew of 1.47. Similarly, the mean serum PSA was 4.21 ± 3.85, with a positive skew of 1.94. Lastly, the median IPSS score was 19 with a mean of 18.89 ± 4.23. Table 2 outlines the frequency distribution of study variables.

**TABLE 2:** A frequency distribution of prostate volume, International Prostate Symptom Score and prostate specific antigen.

Variable	Mean	Standard deviation	Median	Range	Skew
Prostate volume	61.04	21.95	55.5	25.8–135.0	1.47
Serum PSA	4.21	3.85	2.8	0.2–18.4	1.94
IPSS	18.89	4.23	19.0	6.0–31.0	−0.30

PSA, prostate specific antigen; IPSS, International Prostate Symptom Score.

**FIGURE 1:**
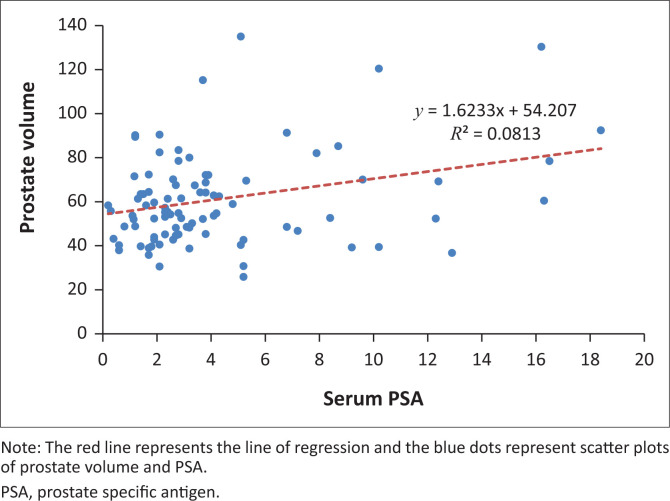
A scatter diagram of prostate volume against serum PSA.

**FIGURE 2:**
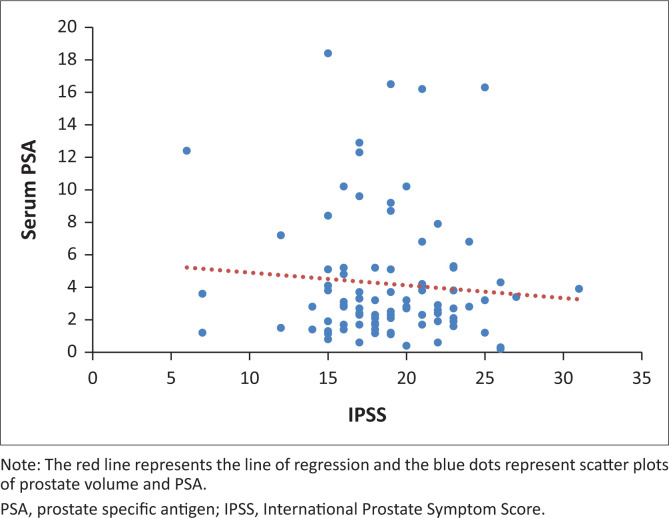
A scatter diagram of serum PSA and IPSS.

**FIGURE 3:**
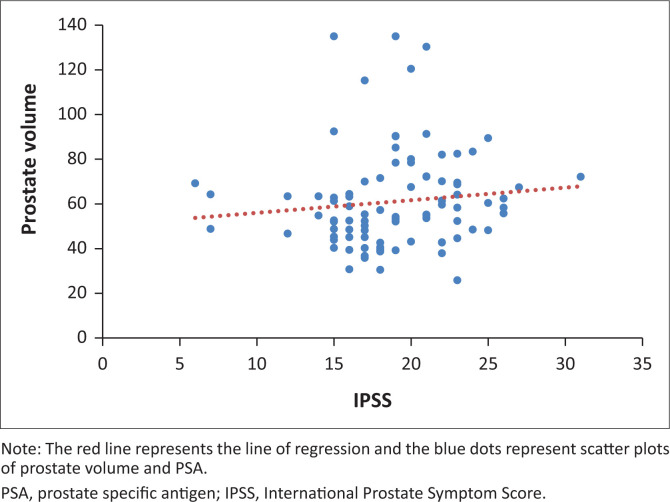
A scatter diagram of prostate volume and IPSS.

When the PV and serum PSA for the 92 patients were subjected to Pearson’s correlation coefficient test, there was a positive significant correlation (*r* = 0.283, *p* = 0.01). Furthermore, when PV and IPSS score were subjected to the same test, there was a positive but statistically insignificant correlation (*r* = 0.108, *p* = 0.30). The evaluation of serum PSA and IPSS showed a negative and statistically insignificant correlation (*r* = −0.086, *p* = 0.42) as shown in the scatter diagrams in Figure 1, Figure 2 and Figure 3.

## Discussion

From this study, PV has been shown to have a weak positive correlation with PSA. International Prostate Symptom Score however had no significant association with PSA or PV. It can therefore be suggested that men with a high PV level will have an increased PSA.

To plan for appropriate urological surgical treatments, surgeons must first determine the size of the prostate gland.^20^ When men of the same age group were studied, the average volume of the prostate was found to vary between communities.^21^ In this study, PV varied widely from 25.8 mL to 135 mL with a median of 55.5 mL. The value of the mean PV (61.04 mL ± 21.95 mL) in this study differed from a similar study on the determination of PV performed by Awaisu and co-workers^22^ on Nigerian men (52.58 mL ± 30.53 mL). However, it was consistent with findings from Turkey^23^ (63.8 ± 30.7) and China^24^ (median = 58.6).

The mean PSA value in this study was 4.21 ng/mL when compared with the normal PSA range it was slightly elevated, a result that differs slightly from other studies. Putra et al.,^25^ Roehrborn et al.^26^ and Mochtar et al.,^27^ obtained 4.61 ng/mL, 2.6 ng/mL, 3.1 ng/mL in their respective studies. Serum PSA has been considered as a PV predictor.^27,28,29^ This is reinforced by the fact that Serum PSA is produced by prostate epithelial cells and studies have found a positive correlation between PSA and PV.^29,30,31,32^ When similar studies were conducted in Japan, India, Indonesia and Nigeria, results were consistent, the corresponding correlation coefficients of these studies were 0.65, 0.78, 0.26 and 0.34, respectively.^25,29,32,33^ This study, however, recorded a weak correlation with a correlation coefficient of 0.28.

This study recorded a mean IPSS value of 18.89 ± 4.29, similar to studies by Agrawal et al.,^13^ Li et al.,^24^ Guzelsoy et al.,^34^ and Malling et al.^35^ who recorded 23.92 ± 6.24, 15 ± 8.4, 17.05 ± 7.62 and 23.5 ± 2.8, respectively. In this study, 42% patients presented with severe symptoms that is IPSS of 20 and above while 55% presented with moderate symptoms, that is, a score of 8–19 and finally, just 3% of patients presented with mild symptoms. This distribution may be because generally, African patients with mild symptoms are unlikely to report to the OPD,^36,37,38^ or are unlikely to be hospitalised unless their quality of life is greatly affected. As such, it was difficult to enrol such patients in this kind of study, which is a usual occurrence in similar study designs.^24,39^ Matoke also found that fear, cost and embarrassment were barriers to seeking treatment for prostatism.^40^

Studies to determine the correlation between PV and IPSS had always yielded varying results. Basawaraj and associates^41^ found a positive correlation between PV and IPSS (*r* = 0.40; *p* = 0.001). However, this is in contrast with several studies that could not establish any statistical significance between the two modalities. For instance, studies carried out in Nigeria and Nepal yielded a correlation coefficient and a *p*-value of *r* = 0.13; *p* = 0.18 and *r* = −0.042; *p* = 0.68, respectively,^15,42^ which implied there was no relationship. The authors obtained a positive correlation coefficient of 0.108 and a *p*-value of 0.30, meaning that there was no association between the two modalities.

Serum PSA is affected by factors such as any pathology of the prostate (prostatitis, BPH and prostate cancer). Even trivial actions such as a digital rectal examination can cause the serum PSA to be elevated. International Prostate Symptom Score on the other hand is a list of questions that are specific in determining the severity of the LUTS, hence the interviewer and the interviewee’s understanding of the set of questions greatly affects the scoring. Coupled with the fact that patients sometimes exaggerate symptoms to receive faster medical attention or understate symptoms in order not to be admitted to the ward, the total IPSS that is obtained from different studies would always have that ‘operator variability’ element, which contributes to why associations vary so much.

Only a few studies have evaluated the relationship between IPSS and PSA. Tsukamoto and colleagues^32^ reported that there was no association between IPSS and PSA (*r* = −0.13; *p* > 0.05) and so did Favilla and associates^43^ (*r* = −0.018; *p* = 0.84). These conclusions however are different from studies by Gnywali and Sharma^42^ and Lim and Buchan^44^ who found positive but weak correlations (*r* = −0.14; *p* < 0.05) and (*r* = −0.04; *p* < 0.05), respectively. This study yielded a negative weak correlation coefficient of −0.09, however, with a *p-*value of 0.42. Therefore, the authors concluded that there was no association between PSA and IPSS.

The use of transabdominal ultrasound for the estimation of the PV might have influenced the PV estimates because it tends to be less reliable than TRUS. Whereas this study established an association between serum PSA and PV, further studies will be required to establish causality. Be that as it may, this study provides baseline data on these clinical parameters in this sub-population.

The authors conclude that that PSA levels have a weak positive correlation with PV, and can also be a reliable indicator of PV in Ghanaian men. The IPSS however had no significant association with PSA or PV. It can therefore be suggested that men with a high PV level will have an increased PSA. But a high PV or PSA does not suggest any bearing on the value of the IPSS or the degree of clinical presentation of LUTS. The authors will therefore caution against the assumption that symptom severity is related to prostate size. Physicians should therefore ensure thorough assessment of patients with prostatism before initiating management.
